# Unintended Pregnancy and Associated Factors among Pregnant Women Attending Antenatal Care Unit in Public Health Facilities of Dire Dawa City, Eastern Ethiopia, 2021

**DOI:** 10.1155/2023/8100462

**Published:** 2023-09-04

**Authors:** Andinet Ayele, Neil Abdurashid, Mickiale Hailu, Bereket Tefera

**Affiliations:** ^1^Action Against Hunger, Addis Ababa, Ethiopia; ^2^Department of Midwifery, College of Medicine and Health Sciences, Dire Dawa University, Dire Dawa, Ethiopia

## Abstract

**Background:**

Unintended pregnancy refers to a pregnancy that is either mistimed or unwanted. Unintended pregnancy has been a troubling public health and reproductive health issue, which imposes appreciable adverse consequences on the mother, child, and the public in general. Globally 121 million unplanned pregnancies occurred from 2015 to 2019. A significant proportion (61%) of these pregnancies ended in abortions each year. In Ethiopia, the challenges of unintended pregnancy and its related complications still exist because of the high rate of unmet need for contraceptives. In addition, no research has been conducted on unintended pregnancy among pregnant women in Dire Dawa city administration.

**Objective:**

To determine the prevalence of unintended pregnancy and associated factors among pregnant women attending antenatal care public health facilities in Dire Dawa in 2021.

**Methods:**

A facility-based cross-sectional study was conducted. After being chosen randomly, 382 pregnant women were interviewed at 9 urban public health facilities. A pretested questionnaire was used to collect data, entered into Epi Info 7, and exported into SPSS version 25 for analysis. The variables, which were significant at *P* ≤ 0.25 in bivariate analysis, were included in multivariable analysis. Statistical significance was declared at a *P* value <0.05 and a 95% CI.

**Results:**

In this study, the prevalence of unintended pregnancy was 23.8% at 95% CI (19.8–28.3). The following factors were associated with unintended pregnancy: single women (AOR = 10.93, 95% CI 3.65–32.74), low family income (2000 ETB) (AOR = 4.01, 95% CI 1.73–9.28), parity 3 (AOR = 10.3, 95% CI 4.07–25.84), no history of family planning use (AOR = 5.91, 95% CI 2.46–14.21), and husband decision-making role on reproductive health (AOR = 2.956, 95% CI 1.048–8.340). *Conclusion and Recommendations*. The prevalence of unintended pregnancy was relatively high in this study. Efforts should be made to scale up women's decision-making power on family planning services and give support to empower women economically. There is the need to promote family planning services to minimize unintended pregnancy and to decrease parity and family size.

## 1. Introduction

Unintended pregnancy refers to a pregnancy that is either mistimed or unwanted. Unintended pregnancy has been a troubling public health and reproductive health issue, which imposes appreciable adverse consequences on the mother, child, and the public in general. Globally 121 million unplanned pregnancies occurred from 2015 to 2019. A significant proportion (61%) of these pregnancies ended in abortions each year. Although there has been a decline in unintended pregnancy around the globe, it has been unequal between high-income countries, compared with middle and low-income countries, where 66 unintended pregnancies per 1,000 women and 93 per 100 women, respectively [1–4].

An analysis of data from a study conducted in developing countries has indicated that the magnitude of unintended pregnancy in those countries varies from 13% to 82%. The results of sub-Saharan multicountry analysis of demographic and health surveys showed that the unintended pregnancy prevalence rate was 29%, ranging from 10.8% in Nigeria to 54.5% in Namibia. In sub-Saharan Africa, unintended pregnancy is still a challenge and remains high due to limited access to reproductive health care. Across sub-Saharan Africa, 35%–65% of pregnancies among human immunodeficiency virus-positive women were unplanned, whereas in Cape Town, South Africa, 46% were reported as unintended pregnancies [5–7].

Unintended pregnancy has serious consequences for women everywhere. Unplanned pregnancy often forces women to confront difficult issues, including abortion, relinquishing a child to adoption, or the raising of a child without the necessary financial, physical, and emotional support. Health consequences include the inherent risks of pregnancy, which may be complicated by other medical problems, and the risks of multiparity. Hemorrhage, infection, and hypertensive disorders, all play a major role in maternal mortality throughout the developing world. Each unintended pregnancy may put women at risk for significant morbidity and mortality due to poverty, malnutrition, lack of health care, and inadequately trained health care providers [5–8].

According to Ethiopia's Mini Demographic and Health Survey 2019, only 41% women use any modern contraceptive method. The recent reports show that the magnitude of unintended pregnancy ranges from 33.3% to 34.8%. Despite the burden of abortion being high in Ethiopia, the unmet need for family planning in the country is still a major reproductive health problem [9, 10].

The induced abortion rate was higher in Dire Dawa and other urban cities as a result of unintended pregnancies [8]. In addition, no information or research has been conducted on unintended pregnancy in the study area (Dire Dawa city administration). For this reason, understanding the prevalence of unintended pregnancy should be pointed out according to the local context, and in addition, identifying factors associated with unintended pregnancy among pregnant women may contribute to the designing of prevention strategies and reproductive health service utilization. Therefore, this study intends to assess the prevalence of unintended pregnancy among pregnant women attending antenatal care units in urban public health facilities in Dire Dawa city.

## 2. Methodology

### 2.1. Study Area, Period, and Design

A facility-based cross-sectional study was conducted among selected pregnant women attending antenatal care units in urban public health facilities in Dire Dawa Administration, Eastern Ethiopia. Dire Dawa city is located 515 km east of Addis Ababa. The administration's total population is estimated to be 506,936 ((urban, 344,716 (68%) and rural, 162,220 (32%)). Reproductive age groups of 15–49 years are estimated at 139,914 (27.6%) and pregnant and lactating women are estimated to be 16,323 (3.22%). The administration has 2 governmental hospitals, 15 health centers, and 34 health posts [11]. The study was conducted from October 15 to November 15, 2021.

### 2.2. Subject of Study and Eligibility Requirements

All pregnant women who were attending ANC units in nine urban public health facilities in Dire Dawa city were the source of the population for the study. Pregnant women who were attending ANC service during the study time period were included in the study.

### 2.3. Sample Size and Sampling Procedure

The sample size was calculated using a single population proportion based on a prevalence of 41.54% reported in Arsi Negele [2]. Then there is the 95% confidence interval (CI *z*/2 = 1.96) and the 5% margin of error (d). The final sample size was 373 with a 10% nonresponse rate and it became 410. However, the total number of pregnant women attending the health care unit was less than 10,000, and we used a correction formula to come up with the final sample size. True sample = (sample size X population)/(sample size + population-1) = (2,259,920)/(5921) = 382.

**Figure 1:**
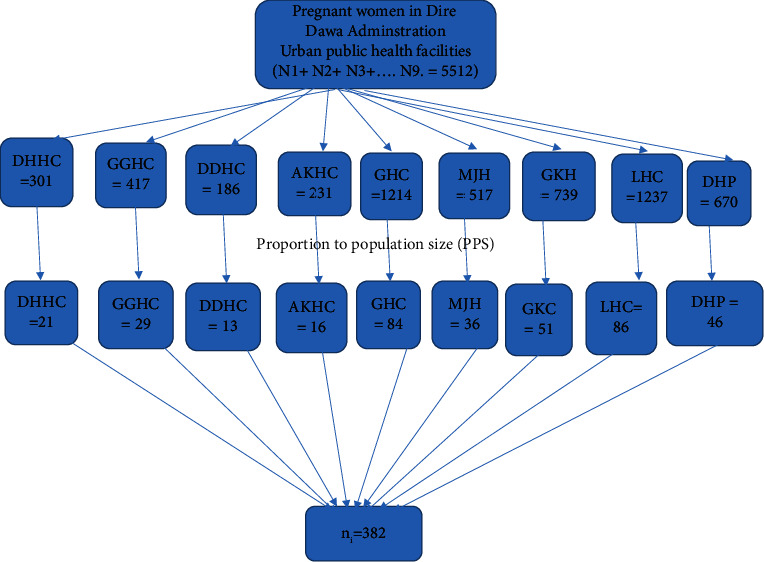
Schematic presentation of sampling procedure for the prevalence of unintended pregnancy and associated factors among pregnant women attending ANC unit at urban public health facilities of Dire Dawa city, 2021. DHHC: Dechatu Health Center, GKHC: Gende Korie Health Center, GGHC: Gendegerada Health Center, LHHC: Legehare Health Center, DDHC: Dire Dawa Health Center,  DHP: Dilchora Hospital, AKHC: Addis Ketema Health Center, PPS: proportional to population size, GHC: Goro Health Center, MJHC: Melka Jebdu Health Center.

Dire Dawa city has eight public PHCUs and two hospitals. Of the nine health facilities (8 PHCUs and 1 hospital) that were included in the study, one hospital was excluded because it was the COVID-19 treatment center. The sample size was proportionally allocated to the selected health institutions. Then, the study participants were selected by using a simple random sampling technique, as shown in Figure 1.

(1) $$\begin{array}{c} Proportionate\;allocation=\frac{nj=n\;Nj}{N}\text{,} \end{array}$$

where nj is sample size of the *j*th health facility, *n* = *n*1 + *n*2 + *n*3 is the total sample size, Nj is population size of the *j*th health facility, and *N* = *N*1 + *N*2 + *N*3 is the total population size.

### 2.4. Data Collection Tools and Procedures

A face-to-face interview was conducted using a structured questionnaire that was adopted from the related studies [12, 13]. The survey questions were under consideration in terms of sequence, cultural sensitivity, and appropriateness. It had three sections. The first section had 10 items and focused on sociodemographic and economic history; the second section consisted of 12 items and was used to assess sexual and reproductive history; and the third section had 10 items, which were used to measure family planning and reproductive health service needs. Nine data collectors (facilitators) and two supervisors were assigned. The interaction was conducted between one interviewer and the interviewee. The room and the environment were made conducive to maintain their privacy and social distance (2-meter distance) between the study participants and the interviewer in order to prevent COVID-19. Wearing of a face mask, clean gloves, and use of hand sanitizer was ensured.

### 2.5. Dependent Variable

1. Unintended pregnancy

### 2.6. Independent Variables

1. Sociodemographic factors (age, education, marital status, residence, occupation, monthly income, religion, and HIV status)
2. Obstetrics history (age at first pregnancy, number of live children, parity, history of unintended pregnancy, and history of abortion)
3. SRH service history (contraceptive use and selection and family planning knowledge)

### 2.7. Operational Definition

#### 2.7.1. Unintended Pregnancy

A woman will be considered to have an unintended pregnancy if her pregnancy is unwanted and mistimed. The items are presented in a structured single response format. Unwanted and untimely responses from mothers are regarded as an unintended pregnancy, while mothers who respond want to be considered as not having an unintended pregnancy [14].

#### 2.7.2. Unwanted Pregnancy

If a woman did not want to become pregnant at conception or at any time in the future [15].

#### 2.7.3. Mistimed Pregnancy

If a woman did not want to become pregnant at the time of conception but did want to become pregnant in the future [15].

### 2.8. Data Quality Control

The questionnaire was written in English first and then translated into local languages and then back into English to ensure consistency. To ensure the validity and reliability of the questionnaires, a pretest was performed on 5% of the sample. Then, appropriate modification was conducted before actual data collection. Training was also given for data collectors and supervisors. Moreover, the filled questionnaires were checked at the field level, first by the data collectors themselves and then by their respective supervisors on a daily basis. At the entry level, the data were checked carefully for invalid codes, duplicated entries, and missing value of records with due emphasis on the expected quality of data. The internal consistency of questionnaire was measured by Cronbach's alpha, and it was reported to be 0.92, which is above the recommended value of Cronbach's alpha, which is 0.70.

### 2.9. Data Process Management and Analysis

Data were coded and checked for completeness and consistency. The data were entered into Epi Info 7 and exported to SPSS software version 25 for further analysis. Descriptive and summary statistics were analyzed. Bivariate analysis was used to test the association between the independent variables and the outcome variable. All explanatory variables that were associated with the outcome variables in bivariate analysis were included in multivariate logistic regression to determine the independent predictor of unintended pregnancy. The variables, which were significant at *P* ≤ 0.25 in bivariate analysis, were included in multivariable analysis. The direction and strength of statistical association were measured by an odds ratio with a 95% CI. The statistical significance level was set at *P* value = 0.05. Finally, the results were presented in tables and figures, which were followed by an interpretation of the findings.

## 3. Ethical Approval and Consent

Ethical clearance was received from Dire Dawa University College of Medicine and Health Science Research and Ethics Review Committee (RERC). A support letter was written to Dire Dawa health bureau to obtain permission, and communication has been established with the head of the health facilities after presenting letters of support. Informed consent was obtained in writing from every participant prior to the start of this research activity, and to ensure confidentiality, participant names were not used during data collection. This anonymity was explained clearly to all participants.

## 4. Results

A total of 382 currently pregnant women were interviewed, and the response rate was 100%. The majority of respondents, i.e., 372 (97.4%) were residents, and nearly one-third, i.e., 125 (32.7%) of the study participants were between the ages of 25 and 29. The mean age of study participants was 25.79 (SD ± 5.32). Concerning marital status, 347 (90.8%) were married and 26 (6.8%) were not married. Of all, 151 (39.5%) women had no formal education and 98 (25.7%) had primary education. More than half, 213 (56.5%) of the respondents were housewives. Of the majority of respondents, 206 (53.9%) reported a medium level of monthly family income (2,000 to 4,999 ETB) and 120 (31.4%) reported a high level of family income. The mean monthly income of the respondents was 4,063 ETB, as shown in Table 1.

### 4.1. Obstetrics History of Study Participants

**Figure 2:**
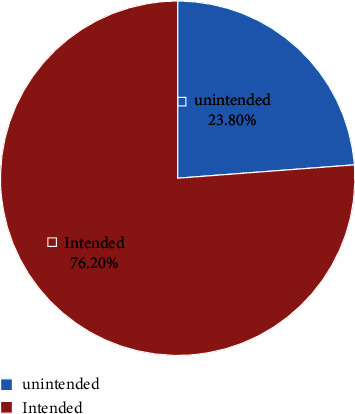
Status of current pregnancy among pregnant women attending ANC unit at urban public health facilities of Dire Dawa administration, 2021.

Three-fourths of the respondents, i.e., 285 (74.6%) had a history of pregnancy, and 176 (46.1%) of the respondents were pregnant for the first time at the age of 15–19 years. Women were asked about the number of pregnancies, 88.8% of the study participants accounted for 1–3 pregnancies and 11.8% of them had 4 or more pregnancies. 222 (58.1%) of study participants have 1–3 children, 118 (30.9%) have no children, and 42 (11%) have more than 3 living children. Regarding the current pregnancy status, 91 (23.8%) of the pregnancies were unintended, of which 55 (14.4%) were mistimed and 36 (9.4%) were unwanted types of pregnancies, as shown in Figure 2.

The remaining 291 (76.2) were intended pregnancies. The most common reasons why they experienced an unintended pregnancy were no use of family planning method 37 (40.7%) and method failure 32 (35.1%). Three hundred thirty-seven (88.2%) had no previous unintended pregnancies but 45 (11.8%) had a previous history of unintended pregnancies. Regarding abortion, 87 (22.8%) of respondents experienced abortion one to two times. Of these, 54 (62%) had experienced spontaneous abortion but 27 (31%) had induced and 6 (7%) had both types of abortion, as shown in Table 2.

### 4.2. Sexual Reproductive Health Service History of Study Participants

Three hundred twenty-four (84.8%) participants responded that they had heard about family planning methods. Health institutions and friends were the main sources of information for family planning, which accounted for 218 (67.3%) and 103 (31.8%), respectively. Nearly one-third of the participants, 135 (41.7%), knew about all methods, while 113 (34.9%) knew only about injectable methods and 45 (13.9%) knew about implants of study participants, and 351 (91.9%) had a history of family planning use. From those who have a history of family planning, the most mentioned method that was reported to be used was injectable (42.7%). Most of the participants, 324 (84.8%), had received information about ANC and PNC as shown in Table 3.

### 4.3. Factors Associated with Unintended Pregnancy among Pregnant Women Attending ANC

In bivariate analysis, marital status, family income, number of pregnancies, number of alive children, decision maker on RH, history of family planning use, and reason not to use family planning were identified to be significantly associated with unintended pregnancy. At multivariate analysis, only marital status, family income, number of living children, decision maker on RH, and history of family planning use remained significantly associated with unintended pregnancy. Marital status was shown to be significantly associated with unintended pregnancy. When compared to married women, single women were about 10.93 times more likely to have an unintended pregnancy (AOR 10.938, 95% CI 3.653–32.749).

In addition, those pregnant women who have a low monthly family income were 4 times more likely to report their current pregnancy as unintended as those women who have a high monthly family income (AOR = 4.017, 95% CI 1.738–9.284).

Unintended pregnancy was ten times more likely (AOR 10.263, 95% CI 4.075–25.849) among pregnant women with four or more alive children than among those with one or two children.

The husband's decision role on the reproductive health of those pregnant women was higher by 2.96 times (AOR 2.956, 95% CI 1.048–8.034) than those women who decided by themselves.

Women who have never used family planning are nearly 5.9 times more likely (AOR 5.915% CI 2.461–14.217) to report an unintended pregnancy as compared to women who have used family planning, as shown in Table 4.

## 5. Discussion

The findings of this study show that 23.8% at 95% CI (19.8–28.3) of study subjects have an unintended pregnancy. Of these unintended pregnancies, 55 (14.4%) of them were mistimed and 36 (9.54%) of them were unwanted. This finding was comparable with that of the previous studies conducted at Gondar 20.6% [15], Mizan Tepi 22.3% [16], Debre Birhan 23.5% [17], Saesie Tsaeda Emba 24.9% [18], and Addis Zemen 26.1% [19]. However, it was higher than that of the study conducted at Belesa 13.7% [20], Egypt 15.9% [21], and Iran 19.8% [22]. The difference may be due to the sample size and study design. The sample size for study conducted in Belesa and Egypt were 619 and 827, respectively, which is larger than our study. In addition to that, both the previous studies were community-based studies. But it was also lower than the study conducted in Michew 29.7% [23], Hadiya Zone 36.2% [14], Hawassa 33.7% [24], and Jimma 36.5% [25]. Those variations may be attributed to the difference in the sociodemographic characteristics; some of them were based on community-based studies, time variation, and sample size. In addition to this, the difference may be due to cultural differences, especially with the other mentioned African countries.

The study also revealed that women who are not married (single) were 10.9 times more likely to report having an unintended pregnancy (AOR 10.938 95% CI 3.653–32.749) than married women. Similar studies conducted in Arsi Negele [2], Gelemso [12], Gondar [15], Michew [23], and Dilla University [26] showed that single women were more likely than married women to have had an unintended pregnancy in the first place. It could be that single women might have unplanned sexual activity for reasons other than child bearing that leads them to unintended pregnancies. In terms of income, pregnant women with a low monthly family income were four times more likely than those with a high monthly family income to have an unintended pregnancy (AOR 4.017, 95% CI. (1.738–9.284)). This study had nearly similar findings to studies conducted in Pakistan [27], Malawi [28], Iran [23], and Egypt [21]. Other studies reported that there is no association between income and unintended pregnancy, which could be because of the different study settings or differences in measurement of the socioeconomic variables like Saesi Tsaedaemba, Woreda, Eastern Zone of Tigray [18], and Arba Minch [14]. It was also noted that the number of alive children was a predictor of unintended pregnancy in this study. Pregnant women with a parity of three or above were 10 times more likely to experience an unintended pregnancy than pregnant women with a parity of one or two (AOR 10.263) (95% CI 4.075–25.849). The finding was in line with that of the studies conducted in Gelemso [12], Addis Zemen [19], and Bako Tibe district [1]. In relation to the association of contraceptive use and unintended pregnancy, women who have never used family planning are nearly 6 times more likely (AOR 5.86, 95% CI 2.440–13.865) to have an unintended pregnancy as compared to women who have used family planning. This finding was comparable with the findings in Arisi Negele [2], Addis Ababa [29], and Hawasa [24]. Furthermore, in this study, unintended pregnancy was associated with decision-making on reproductive health, especially on family planning utilization. Women whose reproductive health was decided by their husband were 2.97 times (AOR 2.974, 95% CI 1.067–8.293) more likely to have an unintended pregnancy compared to those who decided by themselves. Similar findings were observed in different studies conducted in Bale zone [30], Tepi general hospital [16], Arba Minch [31], and Bako Tibe district [1].

### 5.1. Strength and Limitation

#### 5.1.1. Strength

- Use of a pretested questionnaire

#### 5.1.2. Limitation

- The findings cannot be generalized to the whole of Dire Dawa administration because the study was set in urban public health facilities only and not included the rural health facilities and other private health facilities. Since the study followed a cross-sectional design, it does not permit the establishment of causality of unintended pregnancy. In addition, for some of the variables social desirable response bias may have appeared since it was collected by health professionals.

## 6. Conclusions

Approximately one-fourth of the study participants reported that their pregnancy was unintended. Unintended pregnancy was significantly associated with marital status, family income, number of alive children, family planning method use, and decision-making role on reproductive health among study subjects at Dire Dawa city urban health facilities. No use of contraceptives, method failure, and partner refusal were among the main reasons mentioned by study participants for unintended pregnancy. This implies that less effort is being made to provide family planning counseling to women and their partners, particularly single women and multipara women.

## 7. Recommendations

To come up with a solution in order to minimize the problem of unintended pregnancy and the reasons mentioned by the study subjects, the following recommendations are forwarded.

### 7.1. To Ethiopian Federal Ministry of Health

The Ministry of Health should enhance family planning access and promote women's empowerment. Health facilities should design programs to train the community to bring sustainable behavioral changes in reproductive health and promote husband and wife communication about FP. Health professionals, voluntary aid associations, and policy makers should design different policies that help women to restrict family sizes (i.e., family planning policy) and give support to empower women economically.

### 7.2. To Dire Dawa Health Bureau

Programs should be designed in order to ensure good behavioral change on family planning methods among women of reproductive age groups and their partners. More energy should be focused on the dissemination of information on proper, consistent, and effective utilization of contraceptives, partner participation, and role since method failure and partner refusal can lead to unintended pregnancy.

### 7.3. To Health Professionals

Health professionals should give due attention to the improvement in the provision of effective reproductive health counseling and quality of care.

#### 7.3.1. Researcher

Further administration-wide studies should be conducted to show a comprehensive picture of unintended pregnancy and its associated factors.

## Figures and Tables

**Table 1:** Sociodemographic characteristics of pregnant women attending ANC unit at urban public health facilities of Dire Dawa city, 2021.

Characteristics		Frequency (*n*_*i*_ = 382)	Percent (%)
Residence address	Urban	372	97.4
	Rural	10	2.6

Age	15–19	47	12.3
	20–24	116	30.4
	25–29	125	32.7
	30–34	62	16.2
	35–39	29	7.6
	40–45	3	0.8

Religion	Orthodox	131	34.3
	Muslim	223	58.4
	Others^*∗*^	28	7.3

Marriage	Married	347	90.8
	Single	26	6.8
	Separated/divorced/widowed	9	2.4

Ethnicity	Amhara	101	26.4
	Oromo	185	48.4
	Somali	50	13.1
	Others^*∗*^	46	12

Educational status	No formal education	151	39.5
	Primary school	98	25.7
	Secondary school	74	19.4
	Certificate and above	59	15.4

Occupational status	House wife	213	56.5
	Government/NGO employee	61	16
	Daily laborer	28	7.3
	Private business	64	16.8
	Others^*∗∗∗*^	13	3.4

Family income	Low (<2000 ETB)	56	14.7
	Medium (2000–4999 ETB)	206	53.9
	High (>5000 ETB)	120	31.4

HIV status	Positive	13	3.4
	Negative	367	96.1
	Unknown	2	0.5

**Table 2:** Obstetrics history of pregnant women attending ANC unit at urban public health facilities of Dire Dawa city, 2021.

Characteristics		Frequency (*n*_*i*_ = 382)	Percent (%)
History of previous pregnancy	Yes	285	74.6
	No	97	25.4

Age of first pregnancy	15–19	176	46.1
	20–24	157	41.1
	25–29	46	12
	30–34	3	0.8

No. of pregnancy	Less or equal to 3	337	88.2
	Greater than 3	45	11.8

No. of alive children	No child	118	30.9
	1–3	222	58.1
	>3	42	11

Type of unintended pregnancy	Unwanted	36	14.4
	Mistimed	55	9.4

Reasons of unintended pregnancy	No use of FP method	37	40.7
	Method failure	32	35.1
	Husband/partner pressure	19	20.9
	Others	3	3.3

History of unintended pregnancy	Yes	45	11.8
	No	337	88.2

History of abortion	Yes	87	22.8
	No	295	77.2

Number of abortions	One	74	85
	Two	13	15

Types of abortion	Induced	27	31
	Spontaneous	54	62
	Both type	6	7

Decision maker on RH need	Me only	53	13.9
	Me and my husband	278	72.8
	My husband only	51	13.4

Others = rape.

**Table 3:** Sexual reproductive health service history of pregnant women attending ANC unit at urban public health facilities of Dire Dawa city, 2021.

Characteristics		Frequency (*n*_*i*_ = 382)	Percent (%)
Information heard about FP	Yes	324	84.8
	No	58	15.2

From where information get	Family	2	0.6
	Friend	103	31.8
	Media	1	0.3
	Health institution	218	67.3

Type of FP methods be aware of	Pills	27	8.3
	Injectable	113	34.9
	IUCD	2	0.6
	Implant	45	13.9
	Calendar/rhythm	2	0.6
	All types	135	41.7

History of family planning use	Yes	351	91.9
	No	31	8.1

Type of method used	Pills	94	26.8
	Condom	17	4.8
	Injectable	150	42.7
	IUCD	7	1.9
	Implant	75	21.4
	Calendar/rhythm	8	2.3

Information heard about RH	Yes	324	84.8
	No	58	15.2

On which service you heard	Family planning	92	28.4
	PMTCT	24	7.4
	ANC & PNC	122	37.7
	All	86	26.5

Do health providers provide information on RH?	Yes	369	96.6
	No	13	3.4

**Table 4:** Bivariate and multivariate logistic regression analyses of factors associated with unintended pregnancy of pregnant women attending ANC unit at urban public health facilities of Dire Dawa city, 2021.

Variables		Status of current pregnancy		COR (95% CI)	AOR (95% CI)	*P* value
		Unintended	Intended			
Marital status	Married	70	277	1	1	
	Single	19	7	10.74 (4.34–26.56)^*∗*^	10.93 (3.65–32.74)^*∗∗*^	0.001
	Separated/divorced/widowed	2	7	1.13 (0.23–5.56)	1.09 (0.18–6.32)	0.922

Family income (ETB)	Low (<2000 ETB)	33	23	4.942 (2.495–9.788)^*∗*^	4.01 (1.73–9.28)^*∗∗*^	0.001
	Medium (2000–4999 ETB)	31	175	0.61 (0.344–1.08)	0.57 (0.29–1.12)	0.106
	High (>5000 ETB)	27	93	1	1	

No. of pregnancy	≤3	67	270	1		
	>3	24	21	4.60 (2.41–8.76)^*∗*^		

No. of alive children	No children	26	92	1	1	
	1–3	41	181	0.8 (0.46–1.39)	0.80 (0.33–1.91)	0.382
	>3	24	18	4.71 (2.22–9.99)^*∗*^	10.26 (4.07–25.84)^*∗∗*^	0.001

History family planning use	Yes	74	277	1	1	
	No	17	14	4.54 (2.14–9.64)^*∗*^	5.91 (2.46–14.21)^*∗∗*^	0.001

^*∗*^ *P* value <0.25, ^*∗∗*^*P* value <0.05.

## Data Availability

All the datasets for this study are available from the corresponding author upon request.

## Abbreviations

ANC: Antenatal care
AOR: Adjusted odds ratio
COR: Crude odds ratio
ETB: Ethiopian birr
PMCT: Prevention of mother to child
PNC: Postnatal care
RH: Reproductive health.
